# Supplementary material for: Universal in vivo Textural Model for Human Skin based on Optical Coherence Tomograms
Source: Sci Rep. 2017 Dec 20;7:17912. doi: 10.1038/s41598-017-17398-8 (PMC5738372; doi:10.1038/s41598-017-17398-8)
Supplement: Supplementary file 1 — Supplementary Material [file 41598_2017_17398_MOESM1_ESM.pdf]

Universal *in vivo* Textural Model for Human Skin based on Optical Coherence Tomograms

Saba Adabi[1,2], Matin Hosseinzadeh[3], Shahryar Noei[3], Silvia Conforto[2], Steven Daveluy[4, 5], Anne Clayton[1], Darius Mehregan[4,5], Mohammadreza Nasiriavanaki[1,4,5 *]

[1] Biomedical Engineering Department, Wayne State University, Detroit, MI, USA
[2] Applied Electronics Department, Roma Tre University, Rome, Italy
[3] Departments of Electrical Engineering, Sharif University of Technology, Tehran, Iran
[4] Department of Dermatology, Wayne State University School of Medicine, Detroit, MI, USA
[5] Barbara Ann Karmanos Cancer Institute, Detroit, MI, USA

Supplementary materials

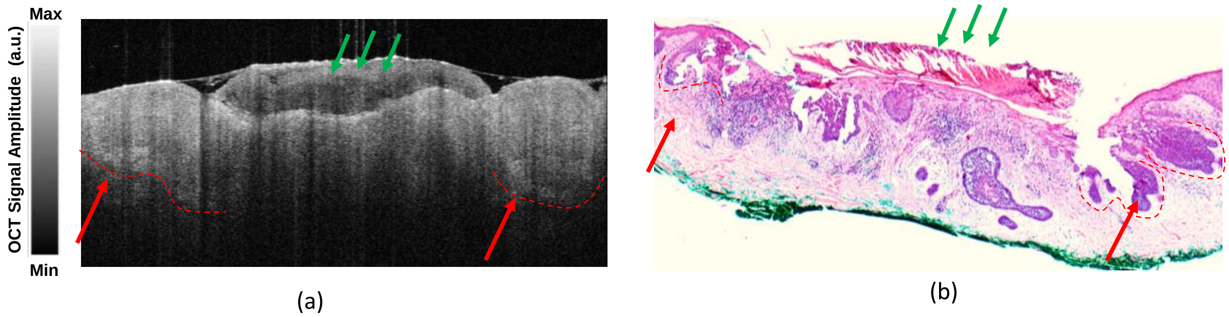

Figure Supplementary S1. (a) OCT B-scan, and (b) its corresponding histological image of a BCC from a 62 year old female. In both OCT and histology images of BCC, the central portion of the epidermis is ulcerated and covered with a crust (green arrow). On either side of the ulceration, there are tumor nodules (red arrows). On the histology images, there are artefactual fractures within the tumor masses that occurred during tissue processing so they are not present in the OCT images.

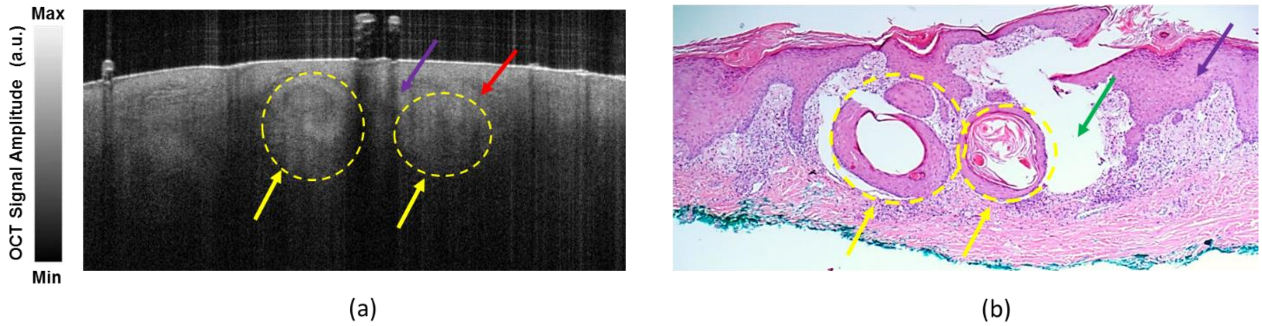

Figure Supplementary S2. (a) OCT B-scan, and (b) its corresponding histological image of an SCC from a 51 year old female. Keratinous pearls within SCC are shown with yellow arrows in both OCT and corresponding histology image. There is a proliferation of keratinocytes in the epidermis pushing into the dermis (red arrow). Keratinocytes in the epidermis show atypia (purple arrow) with large nuclei. The green arrow in the histology image, labels a tear in the tissue that we do not see on the OCT image since it is an artefact of tissue processing. On OCT image, the infiltration of tumor cells into the dermis leads to a loss of the dark line representing the dermo-epidermal junction.

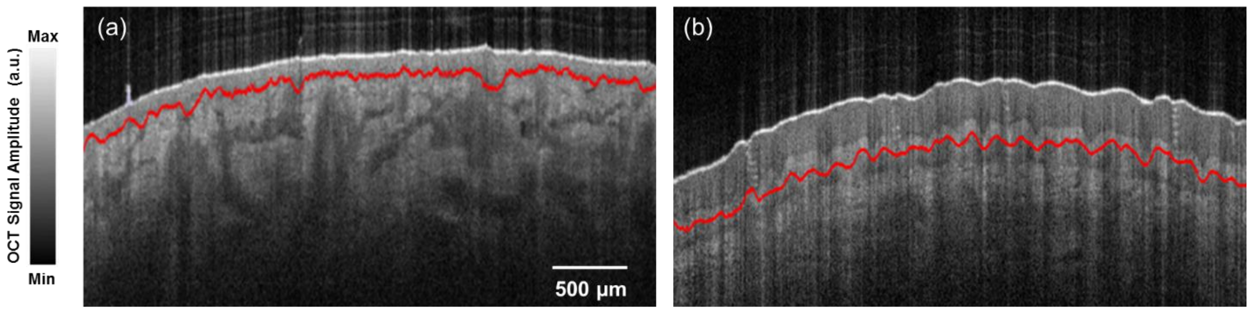

Figure Supplementary S3. Examples of the skin layer detection algorithm applied to OCT images. Application of our DEJ detection method on OCT images of (a) prearicular (thin skin) and (b) palm (thick skin). The red color is the dermal-epidermal junction obtained from our DEJ detection algorithm.

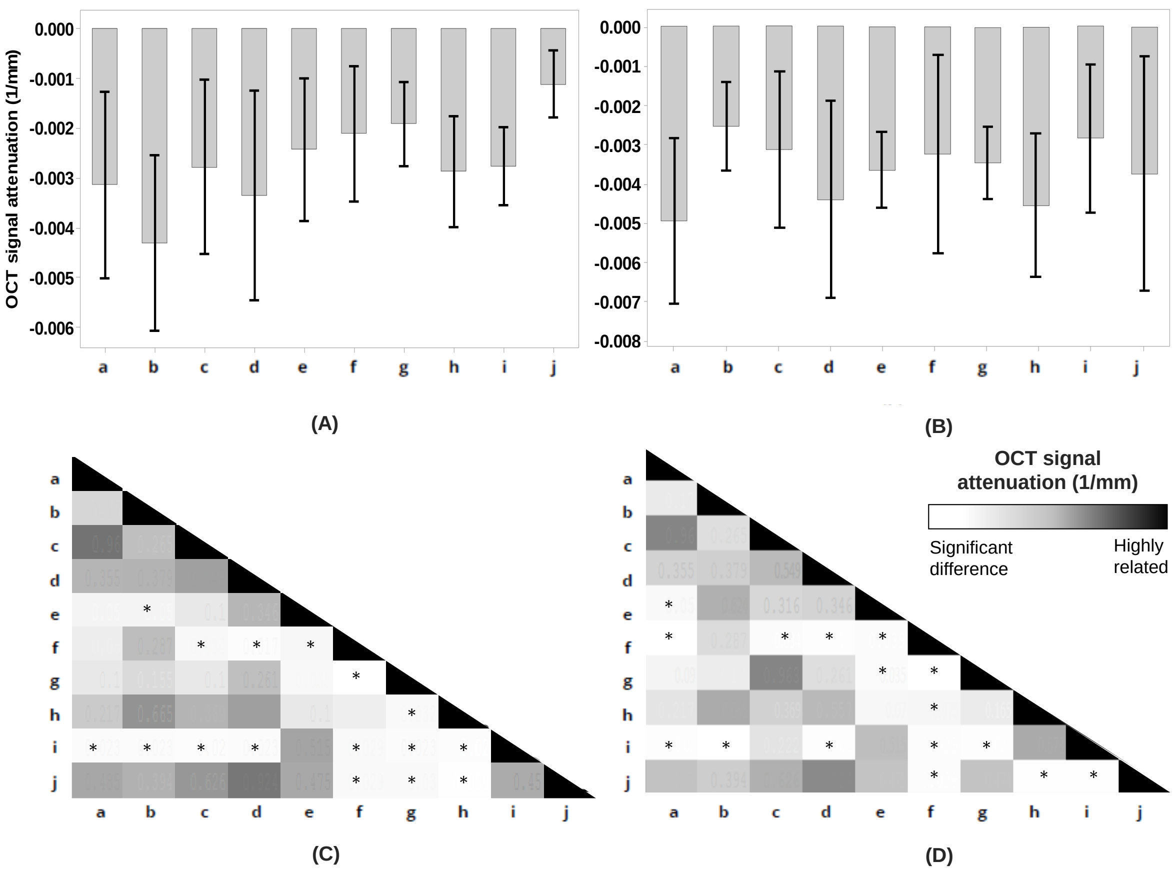

Figure Supplementary S4. Signal attenuation calculated for (A) epidermis, and (B) dermis of ten body sites; Calculated gray-scale coded map of *p*-values between each pairs of body site (*p*-value < 0.05 considered as an acceptable significant difference indicated with *) for (C) epidermis, and (D) dermis. The letters from a-j demonstrate the following; (a) tip of nose, (b) preauricular, (c) volar forearm, (d) neck, (e) palm, (f) back, (g) thumb, (h) dorsal forearm, (i) sole, (j) calf.

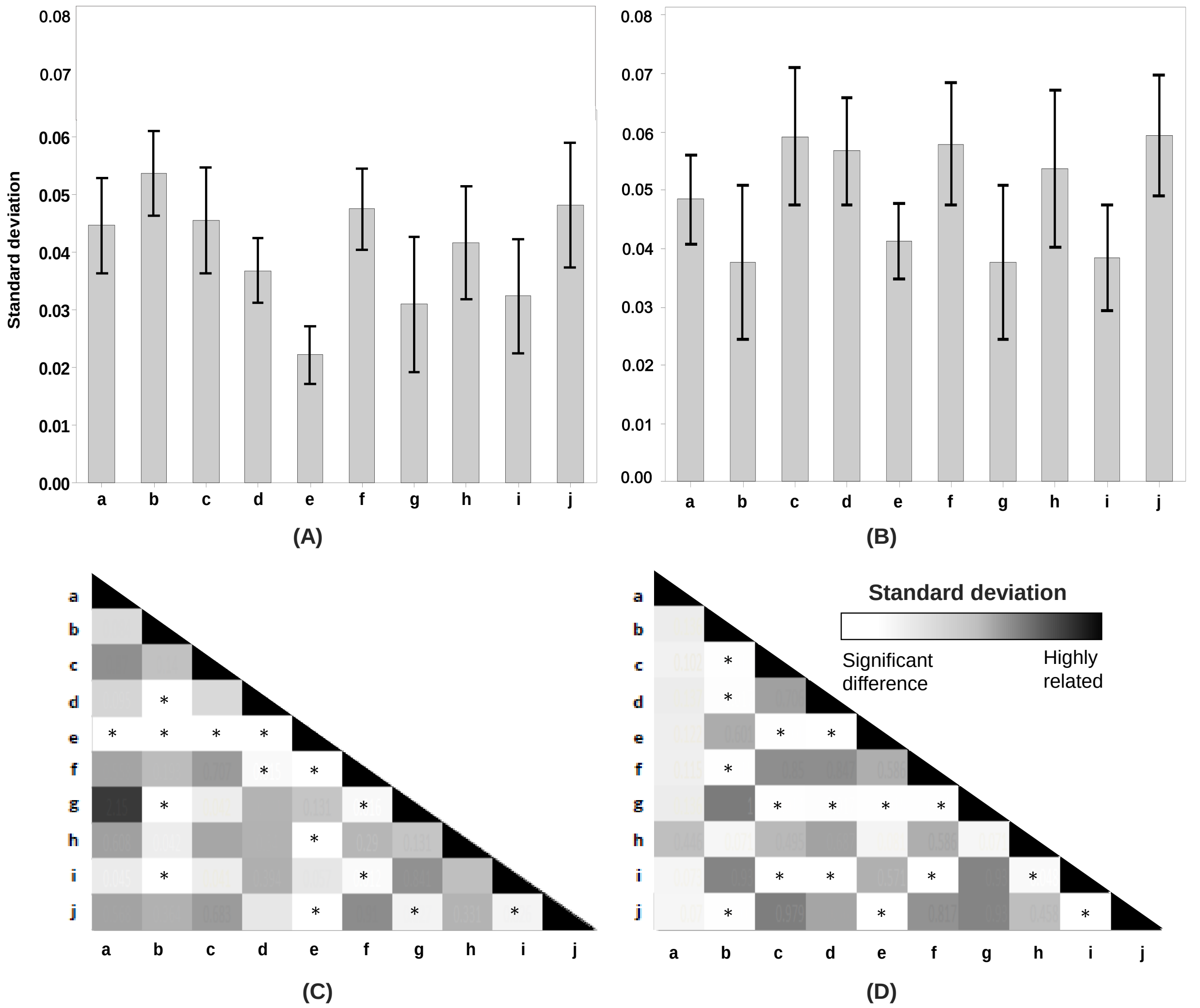

Figure Supplementary S5. First order statistical analysis, standard deviation, results and comparison for (A) epidermis, and (B) dermis of ten body sites; Calculated gray-scale coded map of *p*-values between each pairs of body site (*p*-value < 0.05 considered as an acceptable significant difference indicated with *) for (C) epidermis, and (D) dermis. The letters from a-j demonstrate the following; (a) tip of nose, (b) preauricular, (c) volar forearm, (d) neck, (e) palm, (f) back, (g) thumb, (h) dorsal forearm, (i) sole, (j) calf.

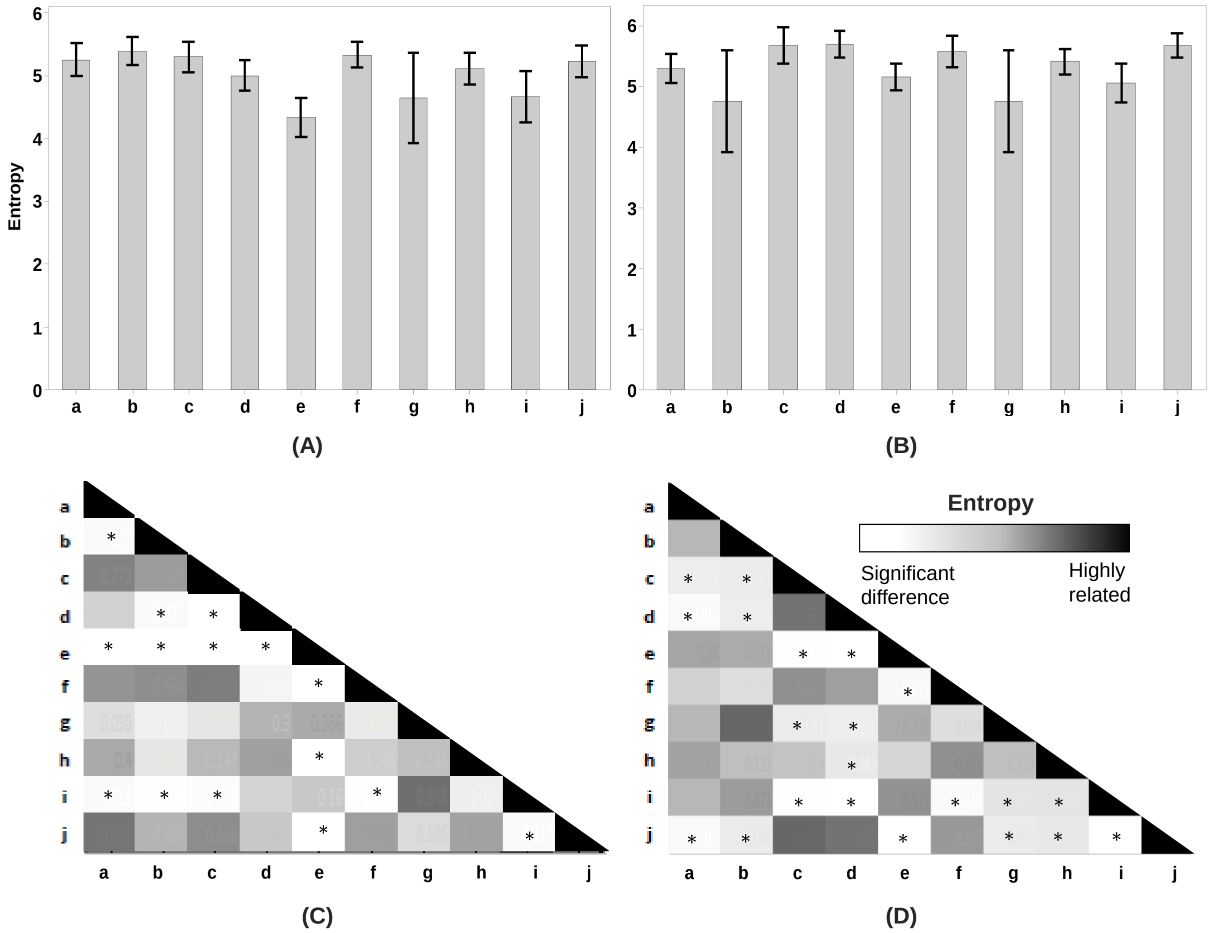

Figure Supplementary S6. First order statistical analysis, entropy, results and comparison for (A) epidermis, and (B) dermis of ten body sites; Calculated gray-scale coded map of *p*-values between each pairs of body site (*p*-value < 0.05 considered as an acceptable significant difference indicated with *) for (C) epidermis, and (D) dermis. The letters from a-j demonstrate the following; (a) tip of nose, (b) preauricular, (c) volar forearm, (d) neck, (e) palm, (f) back, (g) thumb, (h) dorsal forearm, (i) sole, (j) calf.

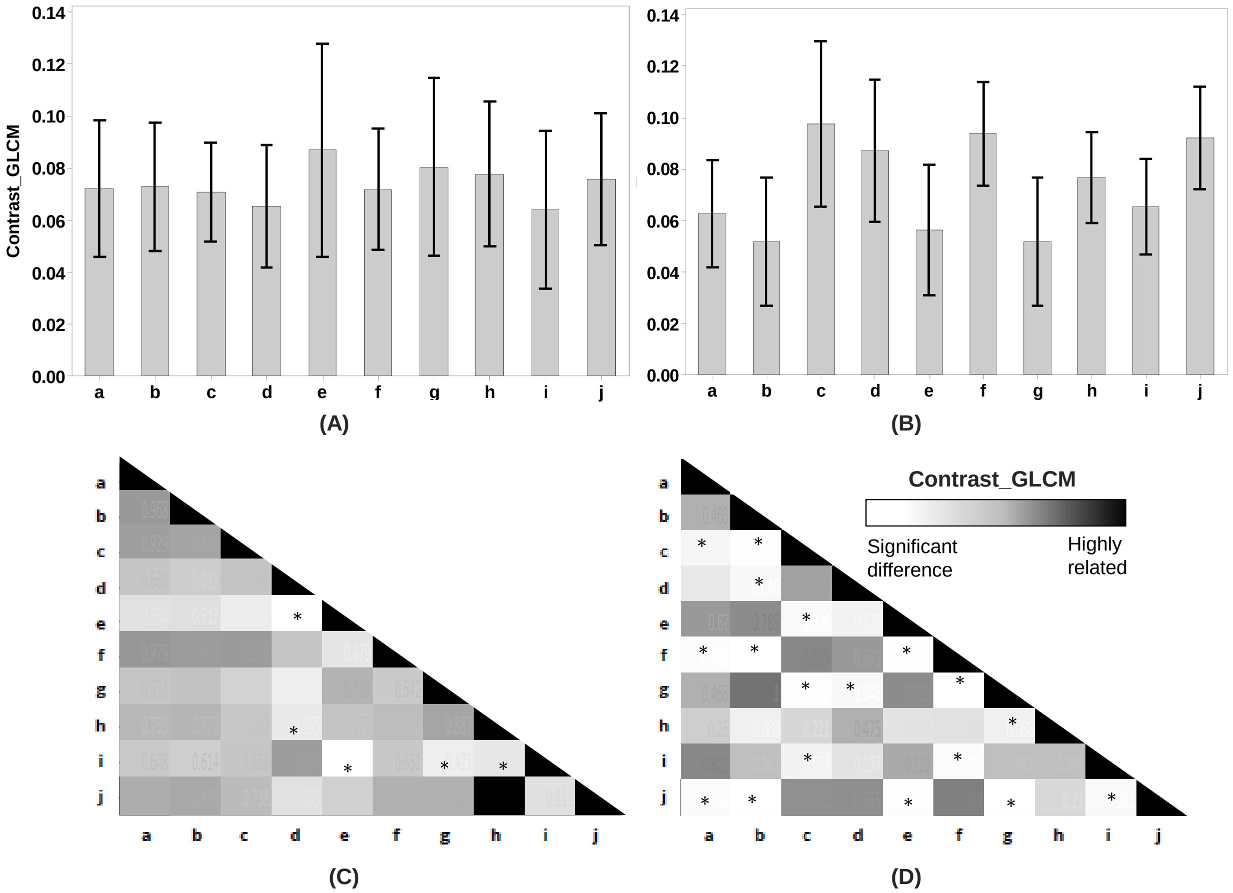

Figure Supplementary S7. GLCM texture analysis, contrast, results and comparison for (A) epidermis, and (B) dermis of ten body sites; Calculated gray-scale coded map of *p*-values between each pairs of body site (*p*-value < 0.05 considered as an acceptable significant difference indicated with *) for (C) epidermis, and (D) dermis. The letters from a-j demonstrate the following; (a) tip of nose, (b) preauricular, (c) volar forearm, (d) neck, (e) palm, (f) back, (g) thumb, (h) dorsal forearm, (i) sole, (j) calf.

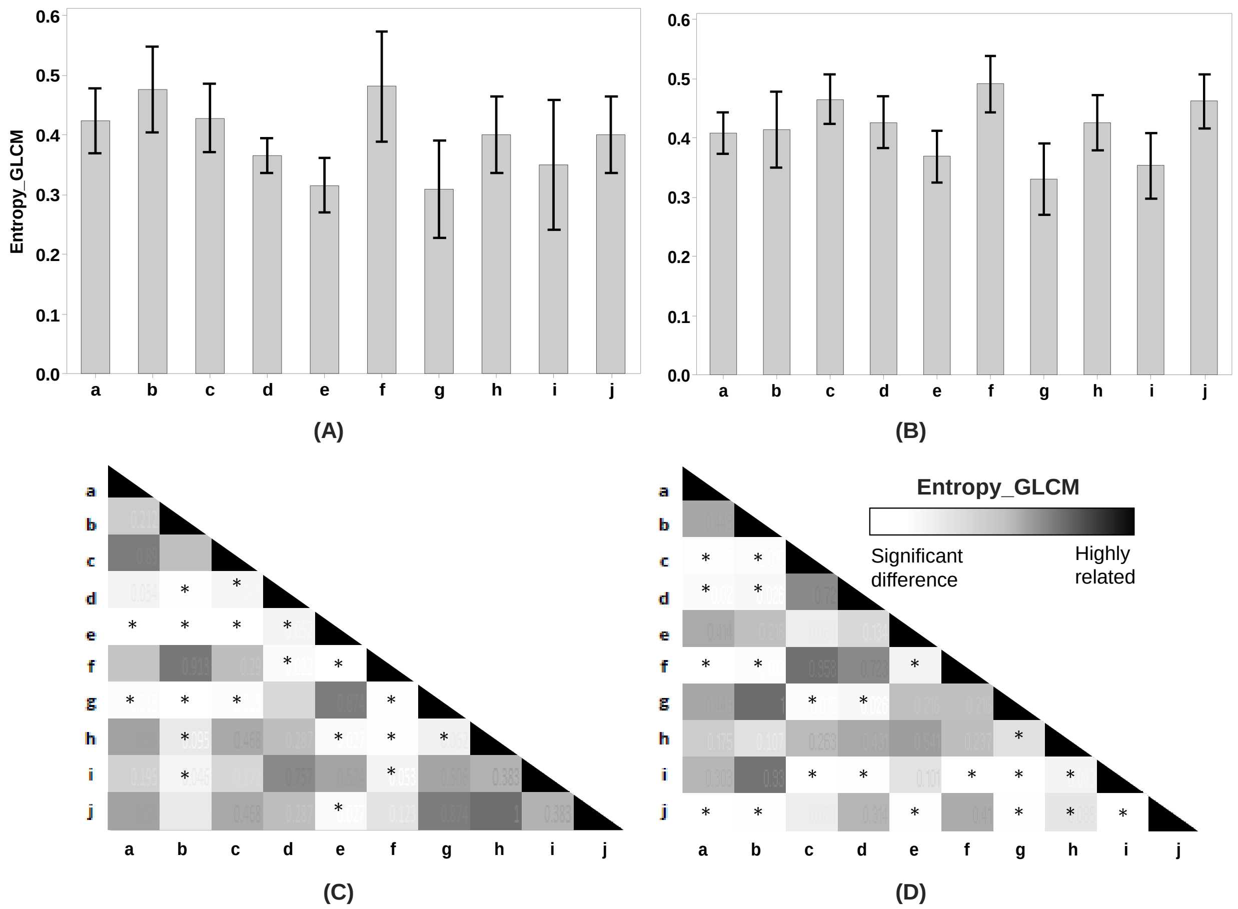

Figure Supplementary S8. GLCM texture analysis, entropy at 45°, results and comparison for (A) epidermis, and (B) dermis of ten body sites; Calculated gray-scale coded map of *p*-values between each pairs of body site (*p*-value < 0.05 considered as an acceptable significant difference indicated with *) for (C) epidermis, and (D) dermis. The letters from a-j demonstrate the following; (a) tip of nose, (b) preauricular, (c) volar forearm, (d) neck, (e) palm, (f) back, (g) thumb, (h) dorsal forearm, (i) sole, (j) calf.

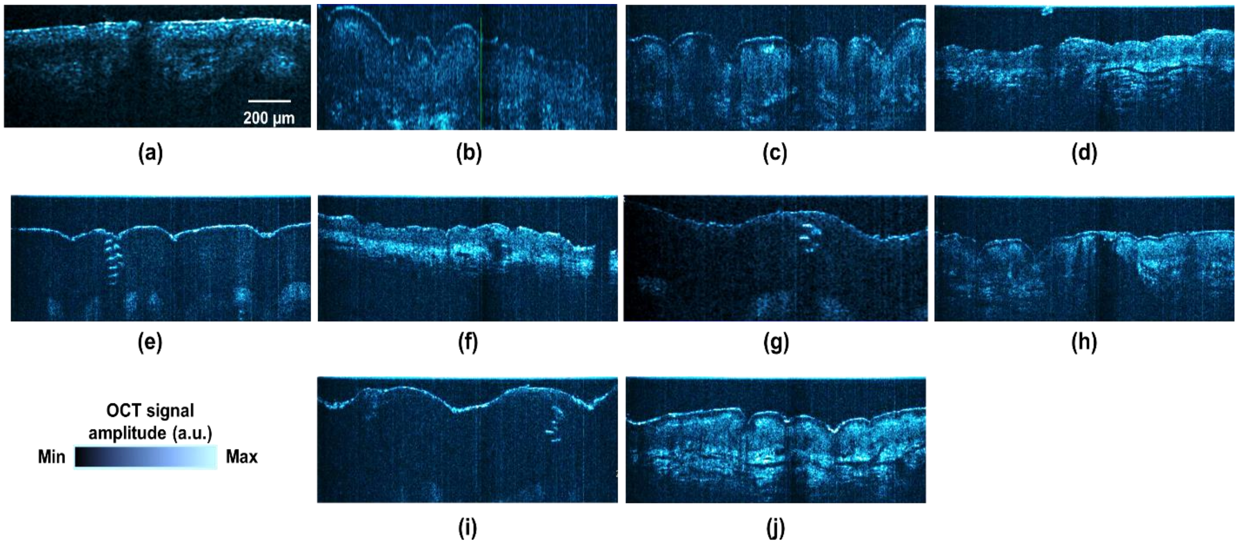

Figure Supplementary S9. HD OCT images of different sites of body including (a) tip of nose, (b) preauricular, (c) volar forearm, (d) neck, (e) palm, (f) back, (g) thumb, (h) dorsal forearm, (i) sole, (j) calf.

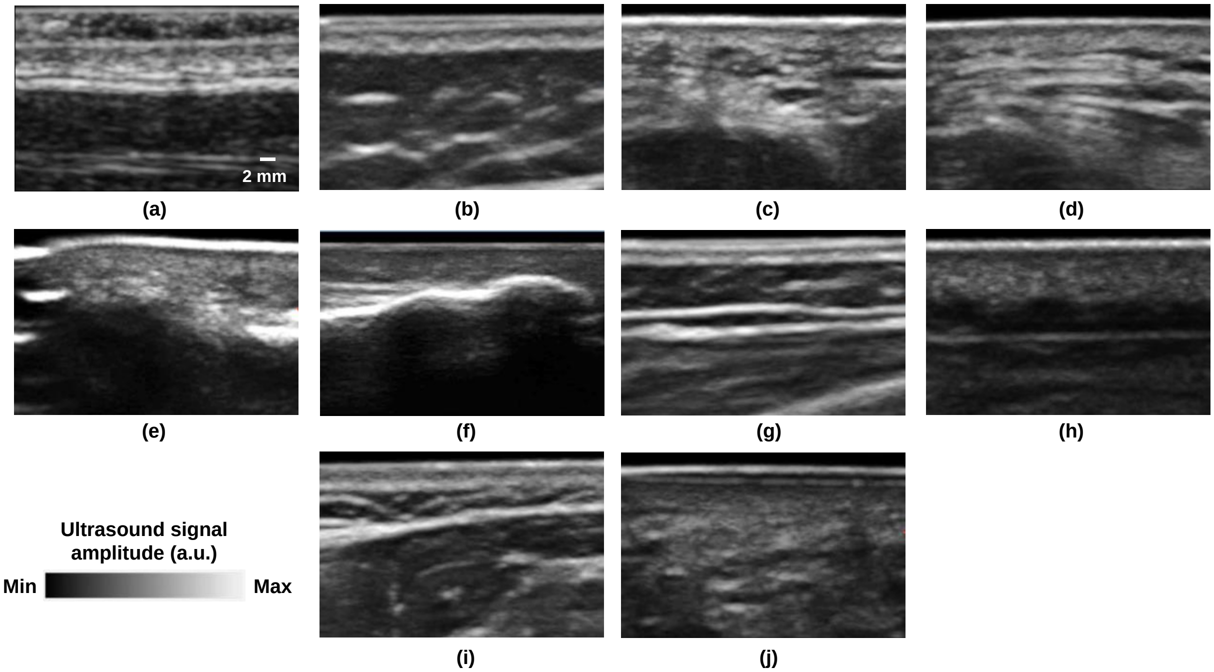

Figure Supplementary S10. Images obtained with a 15 MHz clinical ultrasound probe from different sites of body including (a) tip of nose, (b) preauricular, (c) volar forearm, (d) neck, (e) palm, (f) back, (g) thumb, (h) dorsal forearm, (i) sole, (j) calf.

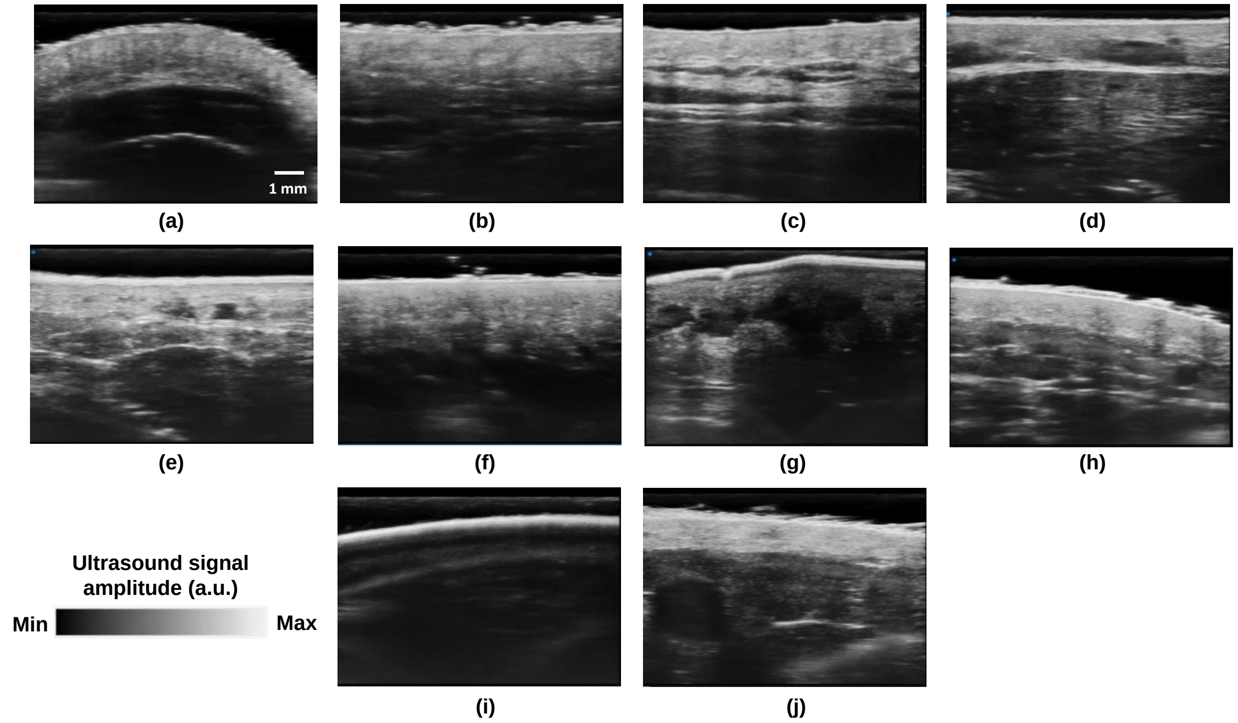

Figure Supplementary S11. Images obtained with a high frequency (VevoMD, 48 MHz) clinical ultrasound probe from different sites of body including (a) tip of nose, (b) preauricular, (c) volar forearm, (d) neck, (e) palm, (f) back, (g) thumb, (h) dorsal forearm, (i) sole, (j) calf.

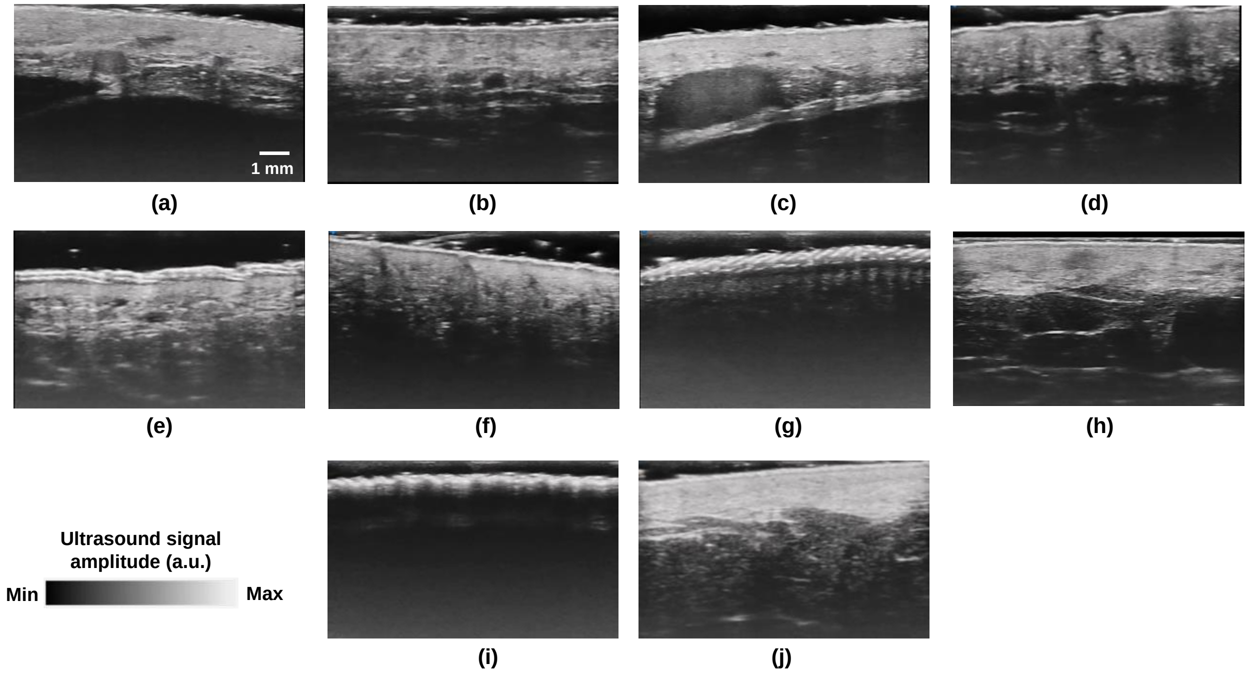

Figure Supplementary S12. Images obtained with a high frequency (VevoMD, 70 MHz) clinical ultrasound probe from different sites of body including (a) tip of nose, (b) preauricular, (c) volar forearm, (d) neck, (e) palm, (f) back, (g) thumb, (h) dorsal forearm, (i) sole, (j) calf.

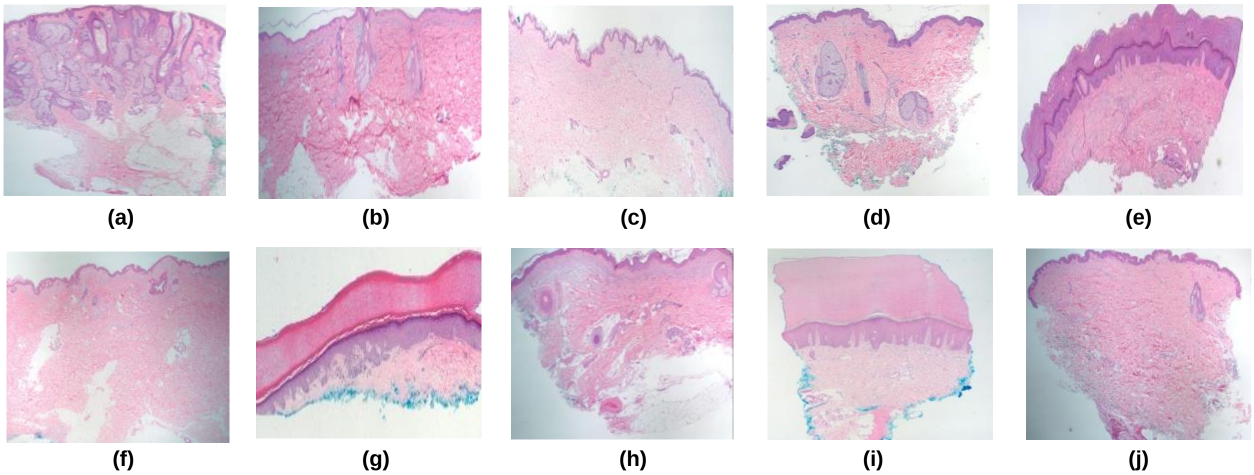

Figure Supplementary S13: istology images of healthy skin at different sites of body including (a) tip of nose, (b) preauricular, (c) volar forearm, (d) neck, (e) palm, (f) back, (g) thumb, (h) dorsal forearm, (i) sole, (j) calf.

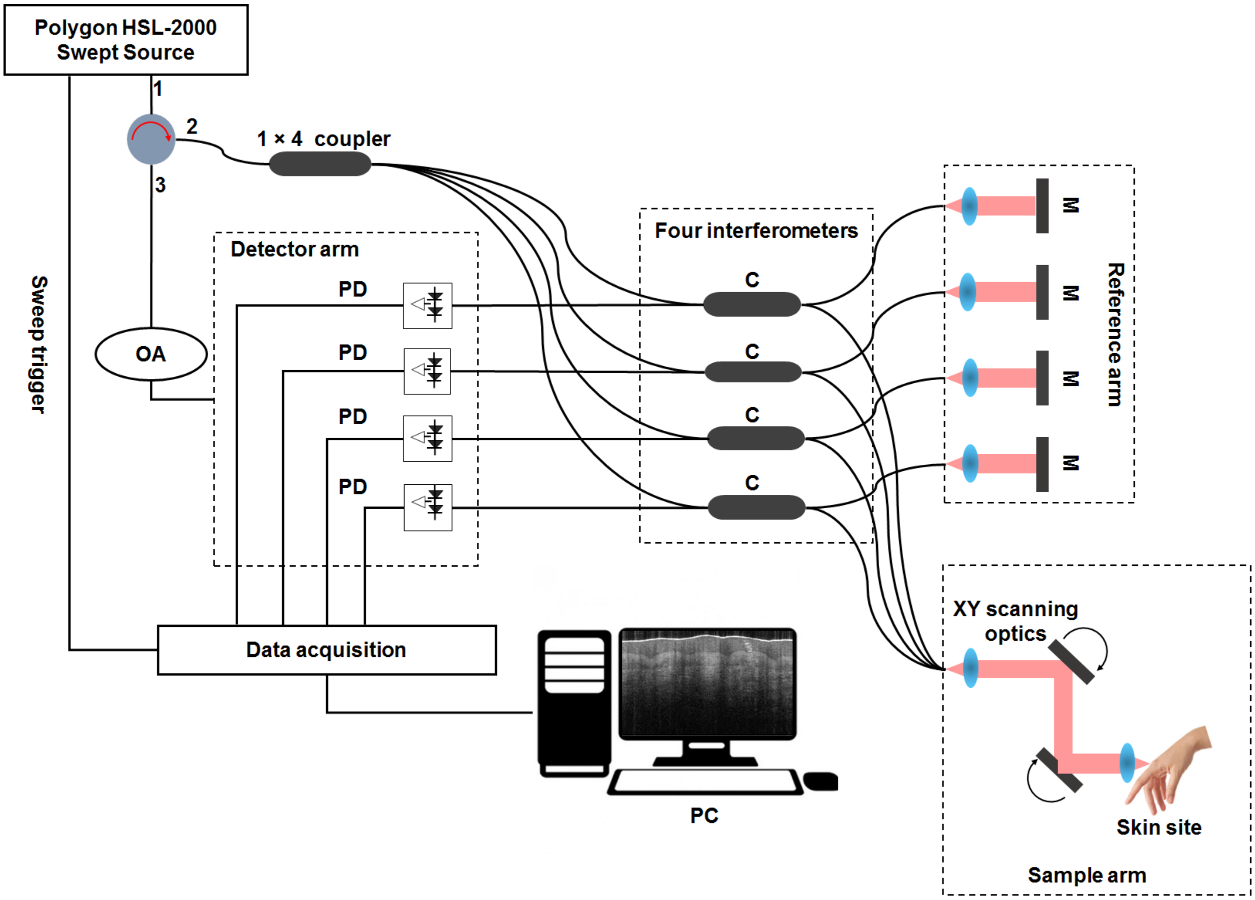

Figure Supplementary S14: Schematic diagram of the multi-beam swept source OCT; M: mirror, C: coupler, PD: photodetector, OA: optical attenuator.
